# Case Report: Omadacycline in the treatment of macrolide-unresponsive *Mycoplasma pneumoniae* pneumonia in an adolescent patient

**DOI:** 10.3389/fcimb.2023.1244398

**Published:** 2023-09-29

**Authors:** Limin Xu, Changquan Fang

**Affiliations:** ^1^ Department of Geriatrics, Huizhou First People’s Hospital, Huizhou, Guangdong, China; ^2^ Department of Pulmonary and Critical Care Medicine, Huizhou Central People’s Hospital, Huizhou, Guangdong, China

**Keywords:** *Mycoplasma pneumoniae* pneumonia, metagenomic next-generation sequencing, omadacycline, macrolide, bronchoalveolar lavage fluid

## Abstract

Omadacycline is a novel tetracycline antibiotic that exhibits good *in vitro* antibacterial activity against atypical pathogens such as *Mycoplasma pneumoniae*. It is approved for the treatment of adults with community-acquired bacterial pneumonia. However, the safety and efficacy of omadacycline in pediatric patients under 18 years of age have not yet been established. In the present paper, we report a case of pediatric community-acquired pneumonia in which initial empirical anti-infective therapy had failed. The patient received empirical anti-infective therapy with azithromycin and other antimicrobial agents upon admission but showed a poor clinical response and developed secondary tinnitus and liver dysfunction. After the confirmation of *M. pneumoniae* infection through metagenomic next-generation sequencing (mNGS) of bronchoalveolar lavage fluid, an antibiotic switch to omadacycline was made. Thereafter, the patient’s condition improved, and no adverse reactions were observed. These findings demonstrate that mNGS enables the identification of infection-causing pathogens in patients with unresponsive pneumonia. Omadacycline can be considered as an alternative option for anti-infective therapy in pediatric *M. pneumoniae* pneumonia, especially when the presence of bacterial resistance, adverse drug reactions, or organ failure are taken into consideration.

## 1 Introduction

*Mycoplasma pneumoniae* is the main pathogen that causes pediatric community-acquired pneumonia. However, given the lack of specificity in clinical manifestations and imaging features and difficulty in obtaining accurate etiological evidence in a timely manner, the diagnosis is often delayed or missed (Huang et al., 2021; Tsai et al., 2021). Research has found that poor outcomes in patients are associated with delayed diagnosis and inappropriate initial treatment. Therefore, early identification of *M. pneumoniae* infection and timely administration of targeted treatment are key to reducing the mortality rate (Tong et al., 2022).

Macrolide antibiotics are currently the first-choice treatment for pediatric *M. pneumoniae* pneumonia. However, there are certain issues associated with these drugs, including bacterial resistance, adverse drug reactions, and restricted use in patients with organ dysfunction (Chen et al., 2020; Vanoverschelde et al., 2021). Omadacycline is a novel semi-synthetic tetracycline that exhibits good *in vitro* antibacterial activity against atypical pathogens such as *Mycoplasma*, *Chlamydia*, and *Legionella*. It is a potential choice for anti-infective therapy in pediatric *M. pneumoniae* pneumonia (Burgos and Rodvold, 2019). However, few cases examining this use have been reported. We present herein a case of pediatric *M. pneumoniae* pneumonia that was unresponsive to initial macrolide treatment but exhibited improvement after treatment with omadacycline.

## 2 Case description

**Figure 1:**
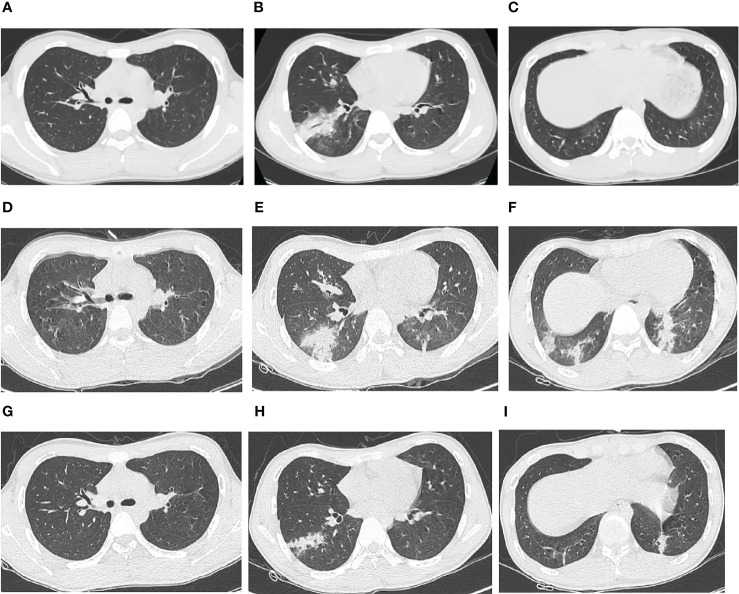
Chest computed tomography (CT) images of an adolescent patient with macrolide-unresponsive *Mycoplasma pneumoniae* pneumonia. **(A–C)** Chest CT 3 days before admission, showing patchy consolidation in the lower lobe of the right lung. **(D–F)** Chest CT on hospital Day 4, showing that the original consolidation in the lower lobe of the right lung had become enlarged and new patchy consolidations had appeared in the bilateral lower lobes. **(G–I)** Chest CT on hospital Day 15, showing significant resolution of the bilateral lung lesions.

A 16-year-old boy was admitted to Huizhou First People’s Hospital on Apr 11, 2022, due to fever and coughing that persisted for 1 week. He developed a fever after cold exposure 1 week prior to admission, with the maximum body temperature being 40.0°C. Other symptoms included chills and headache accompanied by severe paroxysmal cough with a small amount of yellow sputum. The patient self-administered oral cefaclor and paracetamol for 3 days, but the high fever persisted, and the cough worsened. Three days before admission, the patient sought medical consultation at the fever clinic of Huizhou First People’s Hospital. Routine blood work showed a white blood cell (WBC) count of 5.7*10^9/L and neutrophil ratio of 59.1%. Chest computed tomography (CT) revealed patchy consolidation in the lower lobe of the right lung (**Figures 1A–C**). The patient was subsequently hospitalized when intravenous ceftriaxone did not resolve the fever. He had been previously healthy with no history of infectious diseases, such as hepatitis or tuberculosis, recent contact with poultry, or recent travel history.

Physical examination results were as follows: body temperature: 38.2°C; pulse: 72 beats per minute; respiration: 24 breaths/min; blood pressure: 108/70 mmHg (1 mmHg=0.133 kPa). The patient was alert and oriented and provided relevant responses to questioning. No cyanosis in the mouth or lips, yellow discoloration of the skin or sclera, rash, subcutaneous bleeding, or palpable superficial lymph nodes were observed. Coarse breath sounds were heard in both lungs, but neither dry nor moist rales were detected. No other significant abnormalities were observed.

Laboratory test results were as follows: coronavirus disease 2019 RNA and antigen tests, influenza A and B virus antigen tests, and dengue virus antigen test: negative; serum 1,3-β-D-glucan assay, galactomannan assay, Widal test, and Weil-Felix reaction: negative; thyroid function, antinuclear antibody spectrum, and anti-neutrophil cytoplasmic antibodies: negative; sputum, blood, and midstream urine cultures: negative; ELISA for immunoglobulin M antibodies against *Legionella pneumophila*, *M. pneumoniae*, *Chlamydia pneumoniae*, adenovirus, respiratory syncytial virus, influenza A virus, influenza B virus, and parainfluenza virus in the respiratory tract: negative. **Table 1** shows the results of other laboratory tests.

**Table 1:** Laboratory test results at different time points for an adolescent patient with macrolide-unresponsive *Mycoplasma pneumoniae* pneumonia.

Laboratory test	Normal range	First day of hospitalisation	Fourth day of hospitalisation	After 3 days of treatment with omadacycline	One day before discharge
Routine bloodwork					
WBC (×10^9^/L)	4–10	5.7	14.5	6.7	7.3
Neutrophil (%)	40–75	59.1	78.4	64.6	55.7
Inflammatory index					
C-reactive protein (mg/L)	0–5	48.1	65.0	27.1	1.6
Procalcitonin (ng/mL)	0–0.05	0.09	0.25	0.10	0.04
Biochemical indexes					
ALT (U/L)	9–50	24	143	56	31
AST (U/L)	15–40	39	207	81	30
CK (U/L)	50–310	607	885	355	83
LDH (U/L)	109–245	181	396	188	120
D-dimer (mg/L)	0–500	1500	2730	850	340
BUN	3.2–7.1	3.5	4.9	6.0	4.5
Scr (μmol/L)	62–106	76	73	68	76
BNP (pg/mL)	0–100	63.5	75.2	103	10
High-sensitivity troponin T (ng/mL)	14–100	4.8	15.6	11.1	3.8

ALT, alanine aminotransferase; AST, aspartate aminotransferase; BUN, blood urea nitrogen; BNP, brain natriuretic peptide; CK, creatine kinase; LDH, lactate dehydrogenase; Scr, serum creatinine; WBC, white blood cell.

**Figure 2:**
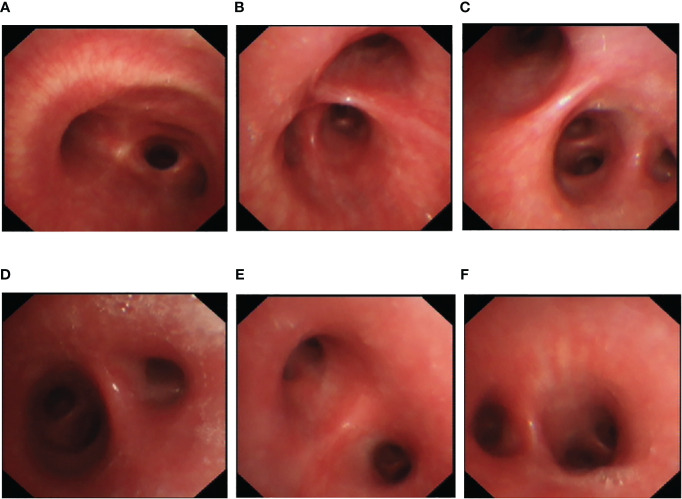
Bronchoscopy findings for an adolescent patient with macrolide-unresponsive *Mycoplasma pneumoniae* pneumonia. **(A–F)** Bronchoscopy shows bronchial mucosa with edema and tracheal hyperemia, with some white secretions in the segmental bronchi.

**Figure 3:**
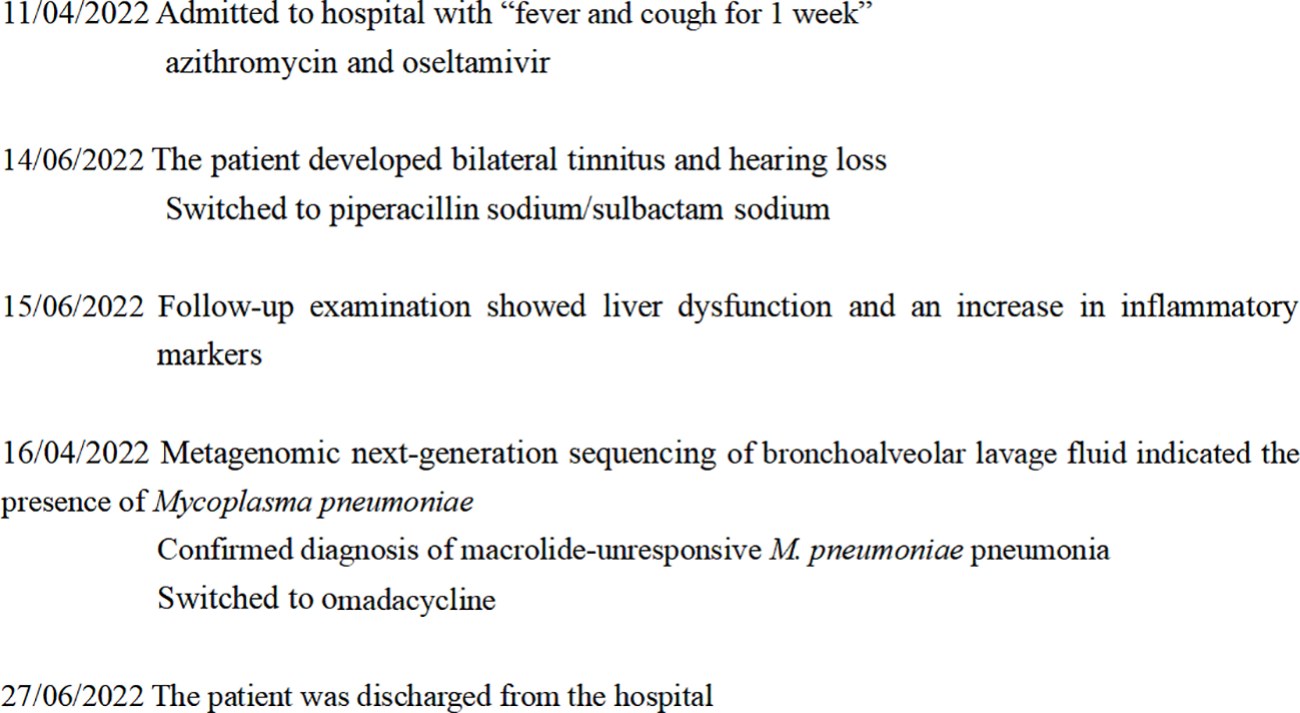
Timeline showing the progress and treatment of macrolide-unresponsive *Mycoplasma pneumoniae* pneumonia in an adolescent patient.

Upon hospitalization, the patient was diagnosed with community-acquired pneumonia. Empirical anti-infective therapy using azithromycin and oseltamivir and symptomatic treatment were concurrently administered. After 72 hours, the patient’s body temperature remained above 38.0°C and the severity of cough and sputum production increased; The expectorated sputum had a white, viscous appearance. The patient also developed bilateral tinnitus and hearing loss. Because adverse reactions to azithromycin could not be excluded, azithromycin was discontinued and piperacillin sodium/sulbactam sodium was administered as anti-infective therapy. On Day 4 of hospitalization, follow-up examination showed liver dysfunction and an increase in inflammatory markers (**Table 1**). Follow-up chest CT showed that the original consolidation in the lower lobe of the right lung had increased in size, with new patchy consolidations in the bilateral lower lobes (**Figures 1D–F**). These findings demonstrated that the initial anti-infective therapy had failed, with the etiology being unclear. Fiberoptic bronchoscopy was performed on Day 4 and revealed the presence of bronchial mucosa with edema and tracheal hyperemia, with some white secretions in the segmental bronchi. (**Figures 2A–F**). The mucus was removed by suctioning, and 10 mL of bronchoalveolar lavage fluid was collected for metagenomic next-generation sequencing (mNGS), which was performed using a DA8600 proton high-throughput sequencing system (Guangdong Ascendas Gene Technology Co., Ltd.; Zhongshan, Guangdong, China). The reference databases for antimicrobial resistance were Comprehensive Antibiotic Resistance Database (CARD) and Antibiotic Resistance Genes Database (ARDB). mNGS results obtained on Day 5 indicated the presence of *M. pneumoniae* (sequence number: 7284, coverage: 99%), 113 sequences of 23SrRNA were detected and no resistance genes were detected, therefore, the 23S rRNA sequence was wild-type. Based on a comprehensive analysis of the patient’s clinical presentations, etiological test results, and response to initial treatment, a diagnosis of macrolide-unresponsive *M. pneumoniae* pneumonia (MUMPP) was made (Chen et al., 2021). As the patient was an adolescent and had developed liver dysfunction, the anti-infective drug was changed to omadacycline (initial dose of 200 mg by intravenous infusion, followed by a dose of 100 mg by intravenous infusion once daily). Three days later, the body temperature returned to normal, and the patient showed an improvement in cough and sputum production. Tinnitus had resolved, and normal hearing had been restored. A follow-up examination indicated a significant improvement in inflammatory markers and various organ function indicators (**Table 1**). The intravenous infusion of omadacycline was continued for 1 week. On Day 15, a follow-up physical examination showed that the inflammatory markers and organ function indicators had returned to normal, and the follow-up chest CT examination revealed significant resolution of the bilateral lung lesions (**Figures 1G–I**). Omadacycline was discontinued, and the patient was discharged the following day (**Figure 3**). One month after discharge, the patient’s general condition was satisfactory with an occasional cough, absence of breathing difficulties and tinnitus, and a normal hearing test result.

## 3 Discussion

*M. pneumoniae* pneumonia, an acute infectious disease of the lower respiratory tract, primarily affects children and adolescents. After entering the human body through the respiratory tract, *M. pneumoniae* can cause direct damage to the respiratory tract epithelium through adhesion and cytotoxic effects. It can also induce pneumonia and other forms of systemic damage through immune mechanisms, with disease severity ranging from asymptomatic to severe. Clinical manifestations, laboratory tests, and imaging characteristics often lack specificity (Kutty et al., 2019; Tsai et al., 2021). The Japan Respiratory Society proposed a clinically based scoring method to aid the rapid diagnosis of *M. pneumoniae* pneumonia based on six criteria: (1) age < 60 years; (2) no or mild comorbidity; (3) paroxysmal cough; (4) no significant abnormalities on lung auscultation; (5) no expectoration or an absence of other pathogens as shown by Gram staining and rapid urine antigen testing; (6) peripheral blood WBC count < 10*10^9/L. Each criterion carries 1 point. If four of the six criteria or three of the first five criteria are met, there is a possibility of *M. pneumoniae* infection (Miyashita et al., 2006; Yin et al., 2012). Five out of these six criteria were met in the present case, confirming that the Japan Respiratory Society rapid scoring method enables early identification of *M. pneumoniae* infection. The imaging presentations of *M. pneumoniae* pneumonia are complex and varied. Characteristic features include bronchovascular bundle thickening, centrilobular nodules, and ground-glass opacities. However, lung consolidations, which are often overlooked, are the only imaging manifestation in certain patients (Miyashita et al., 2009; Izumikawa et al., 2014). In the present case, the fact that lung consolidation was the main imaging presentation and that other characteristic signs of *M. pneumoniae* pneumonia were absent may have been the major reason for delayed diagnosis.

Currently, the diagnosis of *M. pneumoniae* infection primarily relies on serological and polymerase chain reaction (PCR) testing. However, serological tests are time-consuming and cannot provide an early diagnosis, which make them more suitable for retrospective analysis. PCR is highly sensitive and specific; however, it may yield false-positive or false-negative results (Leal et al., 2020; Tang et al., 2021). mNGS is a high-throughput sequencing technology that enables rapid and unbiased detection of various pathogenic microorganisms through shotgun sequencing of nucleic acids from clinical samples (Li et al., 2021). In the present case, initial treatment failed, which led to the suspicion of unresponsive pneumonia. mNGS enabled the identification of *M. pneumoniae* infection in a timely manner, thereby providing a direction for precise anti-infective therapy and avoiding further deterioration of the patient’s condition and antimicrobial misuse. A study by Wang et al. (Wang et al., 2020) on children with severe unresponsive pneumonia showed mNGS provided better sensitivity and accuracy than conventional microbiological test methods. In general, MUMPP was related to mutations in the 23S ribosomal RNA (rRNA) sequence at potential macrolide binding sites (Tong et al., 2022), however, the presence or absence of 23SrRNA sequence mutations were not directly related to MUMPP (Chen, D. et al., 2021). Although no rRNA gene mutations were detected using CARD and ARDB by mNGS, the patient’s condition did not improve after 3 days of treatment with macrolides, and the diagnosis of MUMPP was valid.

Macrolides, fluoroquinolones, and tetracyclines are commonly used drugs in the clinical treatment of *M. pneumoniae* pneumonia (Kutty et al., 2019). Research has shown that *M. pneumoniae* possesses high resistance to macrolides and is sensitive to fluoroquinolones and tetracyclines (Yin et al., 2017; Wang et al., 2022). However, fluoroquinolones are not recommended for the treatment of pediatric patients, as they may possibly cause cartilage developmental disorders in this population (Chen et al., 2020; Ahn et al., 2021). Therefore, tetracyclines should be the first-line drugs for *M. pneumoniae* infection in children aged >8 years. In the present case, the patient was aged <18 years, and initial treatment with macrolides was ineffective and resulted in deterioration of liver function; therefore, treatment with doxycycline or minocycline was considered inappropriate. After switching to omadacycline, a novel tetracycline antibiotic, the patient’s condition improved without the occurrence of adverse reactions. This suggests omadacycline can be considered an alternative anti-infective agent for the treatment of pediatric *M. pneumoniae* pneumonia. To our knowledge, no similar cases have been reported in the literature thus far. Omadacycline is the first aminomethylcycline antibiotic successfully approved for clinical use. It possesses the ability to resist drug resistance mechanisms in bacteria such as drug efflux and ribosome protection and exhibits excellent *in vitro* antibacterial activity against various pathogens, including atypical pathogens, drug-resistant *Streptococcus pneumoniae*, and methicillin-resistant *Staphylococcus aureus* (Burgos and Rodvold, 2019; Zhanel et al., 2020). Studies have revealed that its therapeutic effects in treating adult community-acquired pneumonia are comparable to those of moxifloxacin (Stets et al., 2019). However, the safety and efficacy of omadacycline in pediatric patients aged 8–18 years remain unclear (Burgos and Rodvold, 2019). *M. pneumoniae* infection and anti-infective drugs have a high tendency to cause liver and kidney impairment in pediatric patients, as the liver and kidney functions of children are not yet fully developed (Meng et al., 2021). Pharmacokinetic studies have shown that omadacycline is widely distributed in most tissues throughout the body after administration, with high plasma concentrations achieved in lung tissue. Dose adjustments are not necessary for elderly patients or patients with liver or kidney dysfunction, and the drug has few drug-drug interactions (Burgos and Rodvold, 2019; Rodvold et al., 2020; Zhanel et al., 2020). Therefore, omadacycline offers significant advantages when used as an anti-infective agent for refractory pediatric *M. pneumoniae* pneumonia, especially in cases complicated with liver or kidney failure.

## 4 Conclusion

The clinical and imaging manifestations of *M. pneumoniae* pneumonia lack specificity. Even with the empirical use of macrolide antibiotics, disease progression may still occur, and the possibility of *M. pneumoniae* infection cannot be ruled out. mNGS can aid the early diagnosis of *M. pneumoniae* infection. Timely diagnosis and use of appropriate antimicrobial drugs can improve patient prognosis.

## Data availability statement

The original contributions presented in the study are included in the article/supplementary materials. Further inquiries can be directed to the corresponding author.

## Ethics statement

The studies involving humans were approved by Huizhou First People’s Hospital. The studies were conducted in accordance with the local legislation and institutional requirements. Written informed consent for participation in this study was provided by the participants’ legal guardians/next of kin. Written informed consent was obtained from the individuals and minors’ legal guardian/next of kin, for the publication of any potentially identifiable images or data included in this article.

## Author contributions

LX and CF conceived the work, interpreted the data, revised the manuscript critically for intellectual content and approved the final version for publication. All authors contributed to the article and approved the submitted version.

## References

[B1] Ahn J. G. Cho H. K. Li D. Choi M. Lee J. Eun B. W. . (2021). Efficacy of tetracyclines and fluoroquinolones for the treatment of macrolide-refractory *Mycoplasma pneumoniae* pneumonia in children: a systematic review and meta-analysis. BMC Infect. Dis. 21 (1), 1003. doi: 10.1186/s12879-021-06508-7 34563128 PMC8465761

[B2] Burgos R. M. Rodvold K. A. (2019). Omadacycline: a novel aminomethylcycline. Infect. Drug Resist. 12, 1895–1915. doi: 10.2147/IDR.S171352 31308710 PMC6613460

[B3] Chen Y. C. Hsu W. Y. Chang T. H. (2020). Macrolide-resistant *Mycoplasma pneumoniae* infections in pediatric community-acquired pneumonia. Emerg. Infect. Dis. 26 (7), 1382–1391. doi: 10.3201/eid2607.200017 32568052 PMC7323531

[B4] Chen J. Qi X. Yin Y. Zhang L. Zhang J. Yuan S. (2021). Effects of minocycline on macrolide-unresponsive *Mycoplasma pneumoniae* pneumonia in children: a single-center retrospective study. Transl. Pediatr. 10 (11), 2997–3004. doi: 10.21037/tp-21-356 34976765 PMC8649588

[B5] Chen D. Zhang N. L. Zhang T. Sun X. M. (2021). Detection of drug-resistance genes of *Mycoplasma pneumoniae* in bronchoalveolar lavage fluid of children with refractory *Mycoplasma pneumoniae* pneumonia. CJCP 23 (7), 707–712. doi: 10.7499/j.issn.1008-8830.2104033 34266528 PMC8292659

[B6] Huang X. Li D. Liu F. Zhao D. Zhu Y. Tang H. (2021). Clinical significance of D-dimer levels in refractory *Mycoplasma pneumoniae* pneumonia. BMC Infect. Dis. 21 (1), 14. doi: 10.1186/s12879-020-05700-5 33407216 PMC7787414

[B7] Izumikawa K. Izumikawa K. Takazono T. Kosai K. Morinaga Y. Nakamura S. . (2014). Clinical features, risk factors and treatment of fulminant *Mycoplasma pneumoniae* pneumonia: a review of the Japanese literature. J. Infect. Chemother. 20, 181–185. doi: 10.1016/j.jiac.2013.09.009 24462437

[B8] Kutty P. K. Jain S. Taylor T. H. Bramley A. M. Diaz M. H. Ampofo K. . (2019). *Mycoplasma pneumoniae* among children hospitalized with community-acquired pneumonia. Clin. Infect. Dis. 68, 5–12. doi: 10.1093/cid/ciy419 29788037 PMC6552676

[B9] Leal S. M. Jr. Totten A. H. Xiao L. Crabb D. M. Ratliff A. Duffy L. B. . (2020). Evaluation of commercial molecular diagnostic methods for detection and determination of macrolide resistance in *Mycoplasma pneumoniae* . J. Clin. Microbiol. 58, e00242–e00220. doi: 10.1128/JCM.00242-20 32269102 PMC7269381

[B10] Li N. Cai Q. Miao Q. Song Z. Fang Y. Hu B. (2021). High-throughput metagenomics for identification of pathogens in the clinical settings. Small Methods 5, 2000792. doi: 10.1002/smtd.202000792 33614906 PMC7883231

[B11] Meng Q. Li N. Yuan L. Gao X. (2021). Analysis of common causes of liver damage among children 12 years and younger in Weifang. J. Int. Med. Res. 49, 3000605211006661. doi: 10.1177/03000605211006661 33827321 PMC8040568

[B12] Miyashita N. Matsushima T. Oka M. Japanese Respiratory Society. (2006). The JRS guidelines for the management of community-acquired pneumonia in adults: an update and new recommendations. Intern. Med. 45, 419–428. doi: 10.2169/internalmedicine.45.1691 16679695

[B13] Miyashita N. Sugiu T. Kawai Y. Oda K. Yamaguchi T. Ouchi K. . (2009). Radiographic features of *Mycoplasma pneumoniae* pneumonia: differential diagnosis and performance timing. BMC Med. Imaging 9, 7. doi: 10.1186/1471-2342-9-7 19400968 PMC2680832

[B14] Rodvold K. A. Burgos R. M. Tan X. Pai M. P. (2020). Omadacycline: a review of the clinical pharmacokinetics and pharmacodynamics. Clin. Pharmacokinet. 59, 409–425. doi: 10.1007/s40262-019-00843-4 31773505

[B15] Stets R. Popescu M. Gonong J. R. Mitha I. Nseir W. Madej A. . (2019). Omadacycline for community-acquired bacterial pneumonia. N Engl. J. Med. 380, 517–527. doi: 10.1056/NEJMoa1800201 30726692

[B16] Tang M. Wang D. Tong X. Wu Y. Zhang J. Zhang L. . (2021). Comparison of different detection methods for *Mycoplasma pneumoniae* infection in children with community-acquired pneumonia. BMC Pediatr. 21 (1), 90. doi: 10.1186/s12887-021-02523-4 33607971 PMC7893926

[B17] Tong L. Huang S. Zheng C. Zhang Y. Chen Z. (2022). Refractory *Mycoplasma pneumoniae* pneumonia in children: early recognition and management. J. Clin. Med. 11, 2824. doi: 10.3390/jcm11102824 35628949 PMC9144103

[B18] Tsai T. A. Tsai C. K. Kuo K. C. Yu H. R. (2021). Rational stepwise approach for *Mycoplasma pneumoniae* pneumonia in children. J. Microbiol. Immunol. Infect. 54, 557–565. doi: 10.1016/j.jmii.2020.10.002 33268306

[B19] Vanoverschelde A. Oosterloo B. C. Ly N. F. Ikram M. A. Goedegebure A. Stricker B. H. . (2021). Macrolide-associated ototoxicity: a cross-sectional and longitudinal study to assess the association of macrolide use with tinnitus and hearing loss. J. Antimicrob. Chemother. 76, 2708–2716. doi: 10.1093/jac/dkab232 34312676 PMC8446930

[B20] Wang H. Lu Z. Bao Y. Yang Y. de Groot R. Dai W. . (2020). Clinical diagnostic application of metagenomic next-generation sequencing in children with severe nonresponding pneumonia. PloS One 15, e0232610. doi: 10.1371/journal.pone.0232610 32497137 PMC7272011

[B21] Wang N. Zhang H. Yin Y. Xu X. Xiao L. Liu Y. (2022). Antimicrobial susceptibility profiles and genetic characteristics of *Mycoplasma pneumoniae* in Shanghai, China, from 2017 to 2019. Infect. Drug Resist. 15, 4443–4452. doi: 10.2147/IDR.S370126 35983294 PMC9379117

[B22] Yin Y. D. Wang R. Zhuo C. Wang H. Wang M. G. Xie C. M. . (2017). Macrolide-resistant *Mycoplasma pneumoniae* prevalence and clinical aspects in adult patients with community-acquired pneumonia in China: a prospective multicenter surveillance study. J. Thorac. Dis. 9, 3774–3781. doi: 10.21037/jtd.2017.09.75 29268385 PMC5723809

[B23] Yin Y. D. Zhao F. Ren L. L. Song S. F. Liu Y. M. Zhang J. Z. . (2012). Evaluation of the Japanese Respiratory Society guidelines for the identification of *Mycoplasma pneumoniae* pneumonia. Respirology 17, 1131–1136. doi: 10.1111/j.1440-1843.2012.02227.x 22805282

[B24] Zhanel G. G. Esquivel J. Zelenitsky S. Lawrence C. K. Adam H. J. Golden A. . (2020). Omadacycline: a novel oral and intravenous aminomethylcycline antibiotic agent. Drugs 80, 285–313. doi: 10.1007/s40265-020-01257-4 31970713

